# Endoscopic closure of a congenital tracheo-esophageal fistula using a through-the-scope suturing device in a young boy

**DOI:** 10.1055/a-2612-3065

**Published:** 2025-07-04

**Authors:** Mark Ellrichmann, Gennadii Ivanov, Ina D. Ellrichmann, Mareike Mumm, Andreas Meinzer, Claudio C. Conrad, Robert Bergholz

**Affiliations:** 1 Interdisciplinary Endoscopy, Medical Department 1, University Hospital Schleswig-Holstein, Campus Kiel, Kiel, Germany; 2 54186 Department of General-, Visceral-, Thoracic- and Pediatric Surgery, University Hospital Schleswig Holstein, Campus Kiel, Kiel, Germany; 3 54186 Department of Pediatric Gastroenterology, Clinical of Pediatrics 1, University Hospital Schleswig-Holstein, Campus Kiel, Kiel, Germany

Tracheoesophageal fistulas (TEF) are a congenital anomaly with an incidence of approximately 1 in 4,000 births. TEF are classified into types A–E, with type E being the most amenable to endoscopic closure ( Fig. 1 ) 1 . Advances in endoscopic techniques have expanded the therapeutic options for managing TEF. Here, we present a case of successful endoscopic closure of a TEF using a novel through-the-scope suturing device (TTS-SD, X-Tack, Boston Scientific) 2 . A 37-month-old boy presented with postprandial coughing and recurrent bronchopulmonary infections. Initial endoscopy revealed a small, non-functional porus without significant TEF. Six months later, the patient’s condition worsened with the diagnosis of a significant type E TEF, located 13 cm from the dental arch. An attempt of clip closure failed after 2 weeks. A fully covered self-expanding metal stent (fcSEMS, 10 × 80mm) was then placed but dislocated within days, another fcSEMS (20 × 80mm) was removed after 1 day due to thoracic pain. Given the narrow esophageal diameter, over-the-scope clips and overstitch devices were not feasible. The novel TTS-SD was employed instead. The fistula was debrided with argon plasma coagulation and brushing ( Fig. 2 ). Four helices were placed 4 mm from the fistula margin in a Z-shaped configuration and secured with a closure plug. Air insufflation confirmed successful closure ( Fig. 3 ). At 2 weeks, the helices were partially detached but the patient was asymptomatic. By six weeks, the helices had fully detached, with no symptoms. At 6 months, follow-up confirmed stable closure without recurrence ( Video 1 ). Endoscopic closure of Type E tracheoesophageal fistula presents a minimally invasive alternative to surgery 3 . The TTS-SD significantly expands the endoscopic therapeutic armamentarium, especially in narrow spaces or distorted anatomy. This case demonstrates the successful closure of a congenital H-fistula with endoscopic techniques, suggesting that endoscopy could be the method of choice for treating Type E fistulas.

**Fig. 1:**
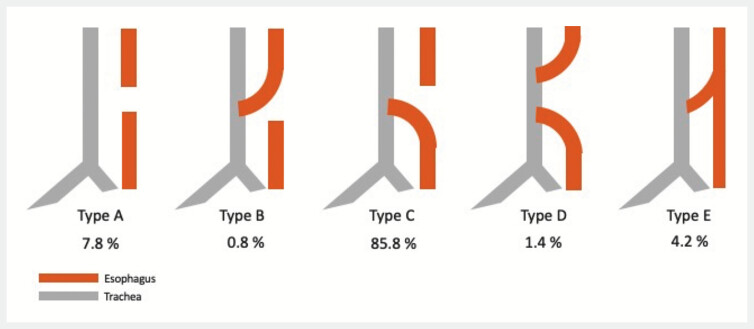
Schematic overview of different categories of tracheo-esophageal fistula Type A–E with respective distribution of occurrence of the subtypes (%), own picture based on McGowan et al. 2022 1 .

**Fig. 2:**
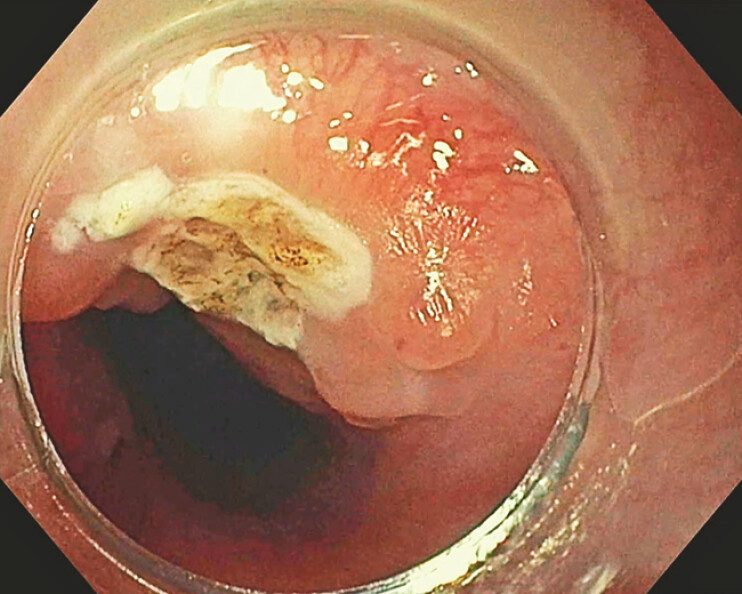
Argonplasma coagulation of tracheo-esophageal fistula Type E.

**Fig. 3:**
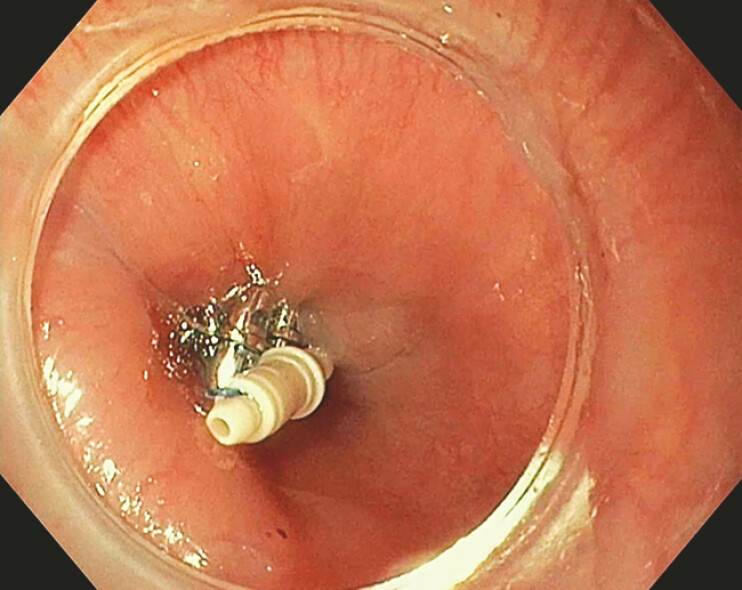
Successful endoscopic suturing of tracheo-esophageal fistula Type E with helices and locking plug in place.

Endoscopic closure of a congenital tracheo-esophageal fistula using a through-the-scope suturing device in a young boy.Video 1

Endoscopy_UCTN_Code_TTT_1AO_2AI

## Notes

Endoscopy E-Videos https://eref.thieme.de/e-videos
*E-Videos* is an open access online section of the journal *Endoscopy* , reporting on interesting cases and new techniques in gastroenterological endoscopy. All papers include a high-quality video and are published with a Creative Commons CC-BY license. Endoscopy E-Videos qualify for HINARI discounts and waivers and eligibility is automatically checked during the submission process. We grant 100% waivers to articles whose corresponding authors are based in Group A countries and 50% waivers to those who are based in Group B countries as classified by Research4Life (see: https://www.research4life.org/access/eligibility/ ). This section has its own submission website at https://mc.manuscriptcentral.com/e-videos .
